# The Radial Propagation of Heat in Strongly Driven Non-Equilibrium Fusion Plasmas

**DOI:** 10.3390/e21020148

**Published:** 2019-02-05

**Authors:** Boudewijn van Milligen, Benjamin Carreras, Luis García, Javier Nicolau

**Affiliations:** 1 Laboratorio Nacional de Fusión, CIEMAT, Av. Complutense 40, 28040 Madrid, Spain; 2 Departamento de Física, Universidad Carlos III de Madrid, Av. de la Universidad 30, 28911 Leganés, Madrid, Spain

**Keywords:** magnetic confinement fusion, turbulence, heat transport

## Abstract

Heat transport is studied in strongly heated fusion plasmas, far from thermodynamic equilibrium. The radial propagation of perturbations is studied using a technique based on the transfer entropy. Three different magnetic confinement devices are studied, and similar results are obtained. “Minor transport barriers” are detected that tend to form near rational magnetic surfaces, thought to be associated with zonal flows. Occasionally, heat transport “jumps” over these barriers, and this “jumping” behavior seems to increase in intensity when the heating power is raised, suggesting an explanation for the ubiquitous phenomenon of “power degradation” observed in magnetically confined plasmas. Reinterpreting the analysis results in terms of a continuous time random walk, “fast” and “slow” transport channels can be discerned. The cited results can partially be understood in the framework of a resistive Magneto-HydroDynamic model. The picture that emerges shows that plasma self-organization and competing transport mechanisms are essential ingredients for a fuller understanding of heat transport in fusion plasmas.

## 1. Introduction

The initial goal of fusion research is to design a system that sustains fusion reactions in a safe manner on Earth, which is a necessary first step towards the development of a fusion reactor, potentially a nearly inexhaustible power source for humankind, free from the pernicious greenhouse effect. Currently, one of the most promising approaches is magnetic confinement, in which the ionized gas or plasma is bound to a strong magnetic field. To avoid end losses, the field lines are bent back on themselves, leading to the typical doughnut-shaped devices called tokamaks and stellarators. The choice of gas is usually a mixture of Deuterium and Tritium, as this combination is easiest to ignite. To achieve sustained fusion reactions, the parameters of the plasma must fulfill the Lawson criterion: nTτ>θ, where *n* is the particle density, *T* the temperature, τ the confinement time, and θ a threshold value [1].

To comply with this requirement in the core region of the plasma, the plasma is heated and fueled by various methods. Without entering into details, we note that temperatures achieved in the core of present-day fusion devices range from about 1000 to several times 10,000 eV, corresponding to equivalent temperatures of 10^7^–10^8^ K. Given such extreme core temperatures, along with the requirement that the walls surrounding the plasma must be kept below the melting temperature of the corresponding materials, it is not unreasonable to state that the temperature gradients created in fusion-grade plasmas are among the highest achieved anywhere on Earth. Hence, the system as a whole is necessarily very far from thermodynamic equilibrium, and standard approaches to study the transport of particles and heat in the plasma must be used with great caution.

Unsurprisingly, the steep gradients, providing an abundance of free energy, trigger the growth of many instabilities, eventually leading to a strongly turbulent state. However, this turbulence is not isotropic, due to the interaction between the dominant confining magnetic field and the ionized plasma, and large-scale coherent structures (known as “zonal flows”, analogous to the bands that form in the atmosphere of Jupiter [2]) tend to form spontaneously, which tame the turbulence somewhat. The ensuing complex multi-scale interactions between turbulence and the large-scale structures often leads to a situation best described as a self-organized state. Due to the existence of thresholds for the triggering of instabilities, it has been surmised that fusion-grade plasmas are, in fact, Self-Organized Critical (SOC) systems, and some evidence has been presented that appears to confirm this conjecture [3].

Since the start of fusion development in the 1950s, progress towards raising the achieved values of the parameters of the Lawson criterion has been steady and rather impressive [4]. However, one issue has kept the fusion community from achieving even higher rates of progress: “power degradation”. Power degradation is the phenomenon whereby the radial outward transport of heat increases more than linearly with the applied input heating power, thus reducing the efficiency of putative fusion power systems significantly. Of course, considering that the system is non-linear and far from equilibrium, it would be somewhat naive to expect this power scaling to be linear. A full understanding of the mechanisms underlying this phenomenon has so far eluded the community.

In the present work, we will address this issue from the novel viewpoint offered by an analysis technique that was recently introduced in the field of information theory: the transfer entropy. This paper is organized as follows. In Section 2, we describe the diagnostic method and the analysis technique used and show a few highlights from the analysis of data from the TJ-I and W7-X stellarators. In Section 3, we show results from the JET tokamak and proceed to analyze these results in more detail, making estimates of “persistency” and an effective diffusion coefficient and interpreting the results in terms of a Continuous Time Random Walk (CTRW). We then discuss this interpretation in light of the simulations of plasma turbulence, which provide some understanding of the reported observations. In Section 4, we discuss our results in the framework of earlier studies and their significance for the power degradation issue. Finally, in Section 5, we summarize our results, which suggest the existence of minor transport barriers and fast and slow heat transport channels.

## 2. Experiments and Methods

Generally speaking, turbulence in fusion plasmas is not easy to study due to the fact that local measurements in the interior of the plasma are difficult to perform. For example, due to the high temperature of the plasma, inserting physical probes is often unpractical and even undesirable due to the induced perturbations. Other measurement systems yield line-integral rather than local quantities (as is the case with some types of electromagnetic emissions from the plasma), generally not very suited to the analysis of turbulence, or only achieve low sampling rates, insufficient to follow the rapid evolution of turbulence in detail (such as the scattering of laser light known as Thomson scattering). Nevertheless, some local and fast measurements are possible. Here, we will focus on a technique known as Electron Cyclotron Emission (ECE).

ECE is a technique developed in the early days of plasma research and is based on a simple physical principle. In the strongly magnetized and highly ionized plasma, electrons gyrate around the field lines with a frequency $\omega_{c}=eB/m_{e}$ and emit radiation at this frequency and higher harmonics. Consequently, the radiation frequency is related to the magnetic field. If the spatial variation of the magnetic field is known, the origin of the emitted radiation can be deduced with good precision, subject to some conditions. The intensity of the detected radiation is directly related to the electron temperature $T_{e}$, again subject to some conditions [5]. Therefore, the measurement of ECE radiation provides a means to study the evolution of the local electron temperature. By measuring at various emission frequencies simultaneously, one may obtain this information at various locations inside the plasma, which is useful to study both the time-averaged temperature profile and the evolution and propagation of temperature perturbations along the measurement chord. Due to these interesting properties of ECE diagnostics, most present-day magnetic confinement devices are fitted with such systems [6].

To probe the transport properties of a system, it is customary to introduce a small perturbation and observe its propagation. The velocity and spreading of the propagating perturbation can then be related to the convection and diffusion coefficients of the system. However, strongly driven fusion plasmas, far from equilibrium, are typically pervaded by many instabilities and noise. Consequently, it is usually not feasible to track individual perturbations, and a statistical approach is needed.

In recent work, we have found that a technique based on ideas from the field of information theory, the transfer entropy, offers a robust way to address this problem [7]. This nonlinear technique measures the “information transfer” or causal relation between two time series. More specifically, the transfer entropy between discretely sampled signals $y(t_{i})$ and $x(t_{i})$ quantifies the number of bits by which the prediction of the next sample of signal *x* can be improved by using the time history of not only the signal *x* itself, but also that of signal *y*.

In this work, we use a simplified version of the transfer entropy:

(1) $$T_{Y\to X}=\sum p\left(x_{n+1},x_{n-k},y_{n-k}\right)\log_{2}\frac{p(x_{n+1}|x_{n-k},y_{n-k})}{p(x_{n+1}|x_{n-k})}.$$

Here, p(a|b) is the probability distribution of *a* conditional on *b*, p(a|b)=p(a,b)/p(b). The probability distributions p(a,b,c,…) are constructed using *m* bins for each argument, i.e., the object p(a,b,c,…) has $m^{d}$ bins, where *d* is the dimension (number of arguments) of *p*. The sum in Equation (1) runs over the corresponding discrete bins. The number *k* can be converted to a “time lag” by multiplying it by the sampling rate. The construction of the probability distributions is done using “course graining”, i.e., a low number of bins (here, m=3), to obtain statistically significant results. For more information on the technique, please refer to [8]. The value of the transfer entropy $T_{Y\to X}$, expressed in bits, can be compared with the total bit range, $\log_{2}m$, equal to the maximum possible value of $T_{Y\to X}$, to help decide whether the transfer entropy is significant or not. The statistical significance of the transfer entropy can be estimated by calculating $T_{Y\to X}$ for two random (noise) signals [9].

The Transfer Entropy (TE) has proven useful for the study of heat transport in stellarators [10,11]. Due to some remarkable properties, the TE is a powerful technique that provides unprecedented radial detail. First, it is directional, acting as a filter that preferentially selects information components related to (directional) propagation. Second, unlike linear tools such as the cross-correlation or the conditional average, it does not depend on the temporal waveform or even the amplitude of the fluctuations, but merely on the time lag between *x* and *y*. A comparison between this technique and the cross-correlation was made in previous work [11], and it was concluded that the TE is an exquisitely sensitive tool to study the propagation of perturbations in highly non-linear systems (such as fusion plasmas), in which perturbations tend to be deformed or change shape quickly as they propagate.

The TE is calculated between two signals, in this case between data measured by an ECE channel at a reference position $r_{\mathrm{ref}}$ (*Y* in Equation (1)) and data from an ECE channel at another position, *r* (*X* in Equation (1)).

**Figure 1:**
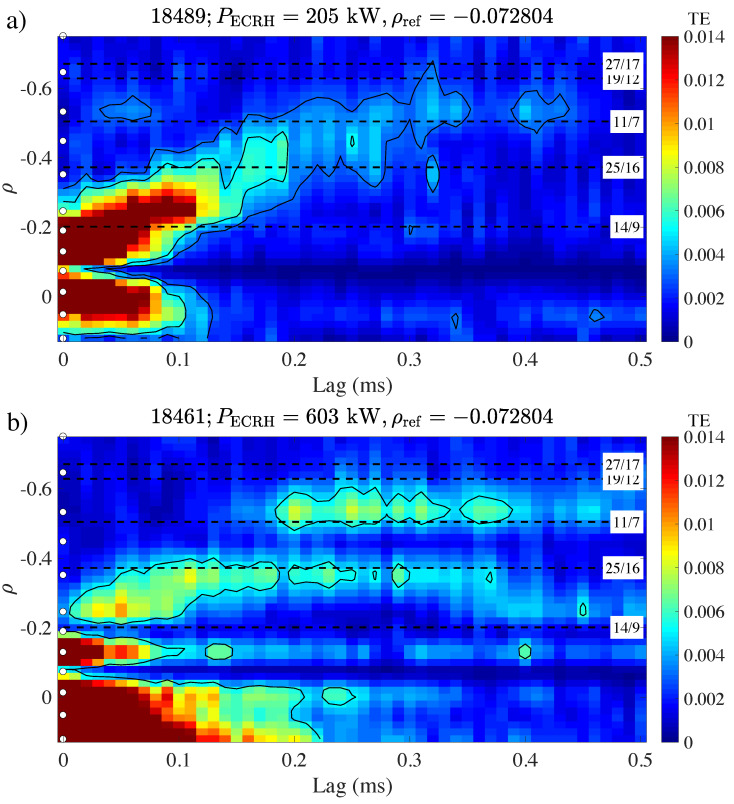
Examples of transfer entropy calculated from Electron Cyclotron Emission (ECE) data taken at the TJ-II stellarator, using $\rho_{\mathrm{ref}}≃-0.07$, at (**a**) $P_{\mathrm{ECRH}}=205$ kW and (**b**) $P_{\mathrm{ECRH}}=603$ kW. The color scale indicates the value of T. ECE channel positions are indicated with white circles. The approximate location of some major rational surfaces is indicated by horizontal dashed lines; the line labels specify the corresponding rotational transform of the magnetic field, n/m (toroidal per poloidal turns). Figure reproduced from [13].

Figure 1 shows an example from the TJ-II stellarator (major radius $R_{0}=1.5$ m) [12], a machine characterized, among other things, by low magnetic shear. The ECE reference channel is taken at $\rho_{\mathrm{ref}}≃-0.07$, and the other ECE channels are distributed along the minor radius −1≤ρ≤1. Here, ρ=0 corresponds to the magnetic axis of the torus, while |ρ|=|r/a|=1 corresponds to the minor radius of the torus. By convention, ECE channels with a negative ρ coordinate (the locations of which are indicated in the figure by white circles) are located on the high field side of the magnetic axis.

The two panels in this figure (a and b) correspond to plasmas with a very different level of electron cyclotron heating power, as indicated in the caption. Comparing the low and high power cases shown in the figure, one observes a relatively smooth “plume” of propagating perturbations in the low-power case, propagating outward from $\rho =\rho_{\mathrm{ref}}$. The main body of the plume occurs in the range −0.35<ρ<−0.07, although a rather weak continuation of the plume reaches about ρ≃−0.55, where some stagnation may be visible. This situation would be roughly consistent with “normal” diffusive propagation. However, in the high-power case, the plume clearly stagnates at ρ≃−0.35, developing a long horizontal “tail”; yet, for τ≃0.2 ms, a second propagation branch appears at ρ≃−0.55, with an amplitude comparable to or greater than the first branch. Note that this response occurs without any detectable response at ρ≃−0.45, so that the perturbations seem to have “jumped over” this intermediate position. The perturbations at ρ≃−0.55 have a stronger causal link to $\rho_{\mathrm{ref}}$ (higher value of TE) than in the low power case. The stronger causal response at ρ≃−0.55 may be related to power degradation, as perturbations seem to be better able to reach this position and influence turbulence there, possibly implying a more intense radial transport from $\rho_{\mathrm{ref}}$ to ρ≃−0.55 in the high power case.

**Figure 2:**
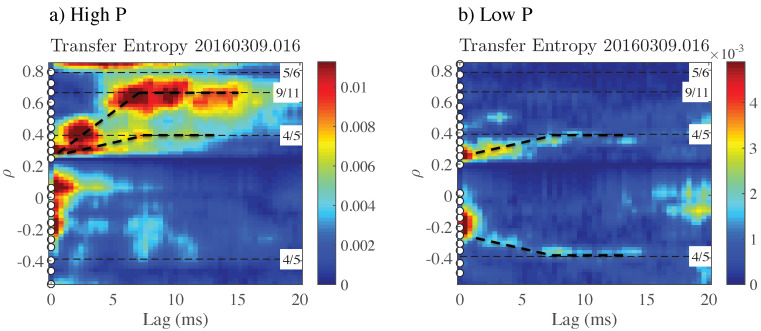
Transfer entropy calculated from ECE data taken at the W7-X stellarator, at (**a**) $P_{\mathrm{ECRH}}≃2.0$ MW and (**b**) $P_{\mathrm{ECRH}}≃0.6$ MW. The color scale indicates the value of T. Radial propagation is indicated with thick dashed lines. Figure reproduced from [11].

Figure 2 shows similar results from a discharge of the W7-X stellarator (major radius $R_{0}=5.5$ m) [14], also with low magnetic shear, but with a size significantly exceeding that of TJ-II. The number of available ECE channels (again indicated by white dots) is much larger here. Note that the convention regarding the radial coordinate, |ρ|=|r/a|, is reversed from TJ-II: here, negative values of ρ correspond to the low field side of the plasma. Due to issues related to data contamination, we only consider data in the range 0<ρ<0.85. Comparing the low and high ECRH power phases, one observes that they have in common that some perturbations propagate outward relatively slowly to the 4/5 rational surface, which acts as a “trapping zone” for these perturbations. In the high power phase, there is an additional branch of radial propagation, faster and more intense (in terms of information transfer), reaching the 9/11 rational surface.

We would like to point out the similarity between Figure 1 and Figure 2. Both show the existence of a clear outward propagating “plume” of “information” from the reference position, $\rho_{\mathrm{ref}}$. This “plume” has a tendency to stagnate near specific low order rational surfaces, producing horizontally extended structures in the figures. On the other hand, occasionally, especially at high power, information is seen to “arrive” at outward positions without having “passed through” positions further inside, giving the impression of having “jumped over” intermediate positions. In the following, we will further investigate this remarkable phenomenology using a different set of techniques.

## 3. Analysis

**Figure 3:**
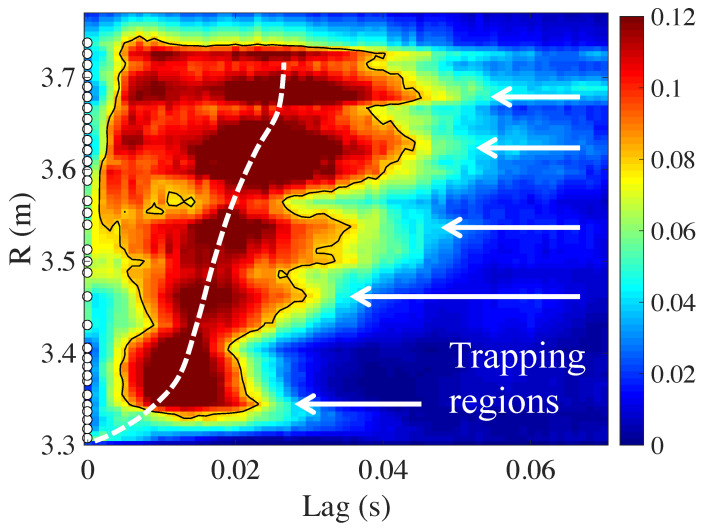
Transfer entropy for JET discharge 82,292. $R_{\mathrm{ref}}=3.30$. The color bar indicates the value of T. White circles indicate the locations of ECE channels. To emphasize the shape of the high TE region, a contour at T=0.08 is shown (black line). The white dashed line indicates the overall outward propagation. White arrows indicate “trapping regions” (see the text). Figure reproduced from [15].

In this section, we will analyze high-resolution ECE data from the JET tokamak (major radius $R_{0}≃2.96$ m) [15]. JET discharges are usually characterized by sawtooth activity in the core region (reconnection events associated with the q=1 rational surface). These events produce a rapid expulsion of heat from the core, and the resulting heat pulses can be analyzed to obtain information about heat transport [16,17,18]. In Figure 3, a typical TE graph is shown for $R_{\mathrm{ref}}=3.30$ m, versus time lag and the *R* value of the other ECE channels. The *R* range is chosen outside the q=1 surface, in order to allow tracking the propagation of the heat pulses caused by the sawtooth crashes. Different from the results shown in Figure 1 and Figure 2, here, the radius indicated on the ordinate of the graph is the major radius, rather than the normalized minor radius. The reader should be aware that the magnetic axis or plasma center is typically located near the major radius of the torus, $R_{0}≃2.96$ m, while the plasma edge is located near R≃3.85 m. This example graph shows that overall transport is outward, as indicated by the white dashed line. The velocity of this propagation, given by the slope of this line, is consistent with the typical heat transport coefficients measured in the JET tokamak using other techniques [19].

### 3.1. Radial Modulation of the TE

We draw attention to the fact that the TE shown in Figure 3 is modulated radially. There are well-defined radial zones where the distribution is broader horizontally than elsewhere, as indicated by the white arrows. As before, we interpret these regions as “trapping regions”, where outward transport is delayed and heat tends to accumulate. Likewise, there are radial “dips” where the TE is significantly lower. In the framework of sheared flow models, “minor transport barriers” are regions where the zonal flow is high and turbulence is suppressed (fully or partially); these regions would correspond to the observed “dips”. The “trapping regions”, however, are zones in-between the minor transport barriers, where turbulence is not suppressed, but turbulent vortices exist that tend to trap the propagating heat.

### 3.2. Persistence of Minima

**Figure 4:**
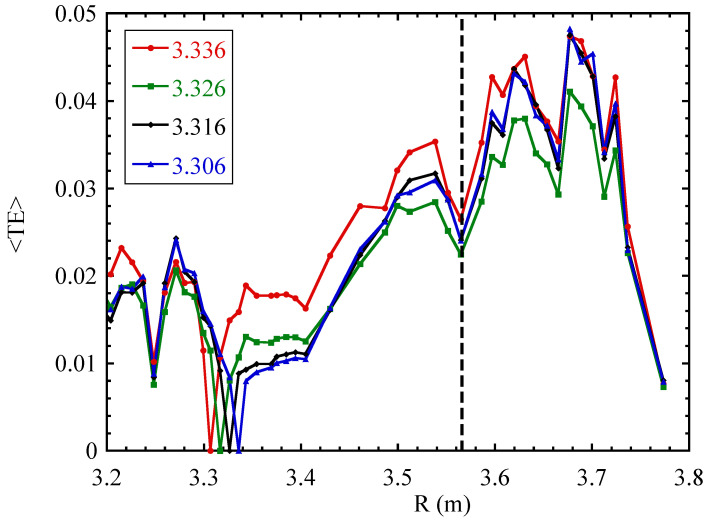
Time average of TE over all time lags 0≤τ≤0.2 s for JET discharge 82,292, for a few reference values $R=R_{\mathrm{ref}}$, as indicated in the legend. Figure reproduced from [15].

In order to quantify the location of the observed radial minima of the TE, we calculate the average of the TE over the available time lags (or up to a specific maximum time lag), 〈T〉. Figure 4 shows an example of this curve for various choices of reference radius. It is observed that the locations of some minima of 〈T〉 do not depend on the choice of reference radius, within a reasonable range, but rather are associated with the magnetic configuration (cf. the minimum indicated by the vertical dashed line in Figure 4). The minimum occurring at the reference radius itself has a trivial origin and should be ignored. The location of minima in the graphs of 〈T〉 can be subjected to a statistical analysis, based on the set of all available $R_{\mathrm{ref}}$ values for a given discharge. To do so, we count how often each local minimum occurs with respect to the total number of reference radii $R_{\mathrm{ref}}$ studied and express it as a percentage. This number is defined as the “persistence” of any given local minimum.

### 3.3. Effective Diffusivity

It is also possible to estimate an effective diffusion coefficient from the radial propagation of information. Calculating an effective diffusion coefficient is important, as it allows contrasting and comparing the results from this method to traditional estimates of heat transport and is helpful to elucidate the power degradation issue mentioned in the Introduction. Nevertheless, it should be borne in mind that the calculation of an effective diffusion coefficient does not imply that transport is actually diffusive in nature; in fact, as we have argued above, it is unlikely to be so. For each available ECE channel, one can estimate the mean time delay 〈τ〉 from:

(2) $$\langle \tau \left(R\right)\rangle =\frac{\int \tau T(R,\tau )d\tau }{\int T(R,\tau )d\tau }$$

**Figure 5:**
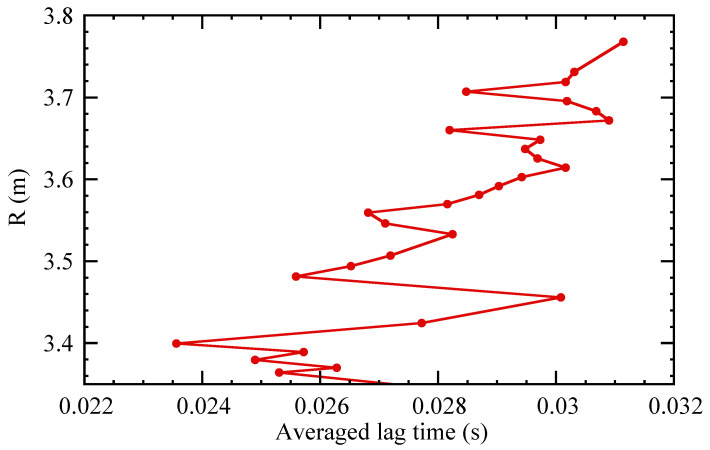
Example graph of position *R* versus the mean lag time 〈τ〉, showing radial variation.

Figure 5 shows an example corresponding to the same case as Figure 4. Using an appropriate reconstruction of the magnetic equilibrium [20], we can convert the ECE measurement location *R* to a minor radius value $r=a\sqrt{\Psi_{N}}$, where $\Psi_{N}$ is the toroidal magnetic flux, normalized such that it equals zero at the magnetic axis and one at the plasma edge (or separatrix).

Then, an effective diffusion coefficient can be defined by:

(3) $$\langle D\rangle =c\cdot \frac{{\left(r-r_{0}\right)}^{2}}{\langle \tau (r)\rangle }$$

The coefficient *c* appearing in this equation is set at $c=\frac{1}{8}$, corresponding to the “time to peak” estimate [21], although slightly different values are sometimes also used in literature [16]. Note that this estimate of the effective diffusion coefficient is not very accurate, for two reasons. First, it is not defined for $r=r_{0}$ as both the numerator and the denominator of the expression tend to zero, and the radial behavior tends to be dominated by the numerator ${\left(r-r_{0}\right)}^{2}$ for small values of $r-r_{0}$. Therefore, the extracted diffusion coefficient should not be taken too seriously in the region near the reference position. Second, it is defined exclusively on the basis of the time (or phase) delay, whereas a proper recovery of the underlying effective diffusion coefficient would require information about the perturbation amplitude as well. Nevertheless, it may serve as a means to visualize the radial variation of transport, and in this paper, we will use it only for this purpose.

The resulting value 〈D〉 is the mean diffusivity over the interval $\left[r_{0},r\right]$. To extract the local value, we consider that this mean diffusivity is calculated as follows from the local diffusivity:

(4) $${\langle D\rangle }_{N}=\frac{1}{r_{N}-r_{0}}\sum_{i=0}^{N-1}\left(r_{i+1}-r_{i}\right)D(r_{i})$$

so that:

(5) $$\left(r_{N}-r_{0}\right){\langle D\rangle }_{N}-\left(r_{N-1}-r_{0}\right){\langle D\rangle }_{N-1}=\left(r_{N}-r_{N-1}\right)D\left(r_{N-1}\right),$$

from which the local effective diffusivity $D(r_{N-1})$ follows. Of course, when 〈D〉 does not depend strongly on *r*, the mean diffusivity and the local diffusivity are nearly the same.

Next, we attempt to correct for the unphysical fact that *D* tends to zero at $r=r_{0}$. To do so, we first compute $D_{0}\left(r\right)$, i.e., the local effective diffusion coefficient using $r_{0}≃0$. Then, we estimate the corrected local effective diffusion coefficient at different reference radii $r_{0}$ using:

(6) $$D_{r_{0}}^{\mathrm{corr}}\left(r\right)=D_{0}\left(r_{0}\right)+D_{r_{0}}\left(r\right)$$

This correction, while still not perfect, should bring the estimated value of the diffusion coefficient closer to the “true” diffusion coefficient, by partially correcting for the unphysical effect mentioned above.

**Figure 6:**
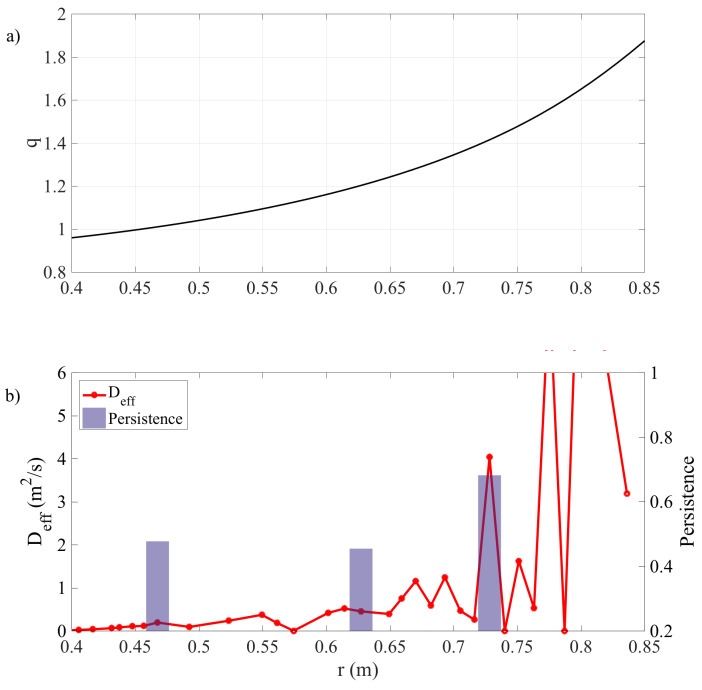
(**a**) Profile of the safety factor, *q*, averaged over the time window of interest (9–12 s) and (**b**) corrected effective diffusion and persistence.

Figure 6b shows an example of the corrected effective diffusion coefficient $D^{\mathrm{corr}}$, along with the location of minima of 〈T〉, indicated by bars proportional to the degree of persistence. It may be observed that structures in the $D^{\mathrm{corr}}$ profile are often correlated with persistent minima, suggesting that these minima indeed act as minor transport barriers, affecting radial heat transport. Figure 6a also shows the corresponding profile of the safety factor, q=m/n (from a reconstruction of the magnetic equilibrium by the program EFIT, using magnetics alone; the sawteeth inversion radius, determined from the $T_{e}$ time traces, is located at r≃0.47, close to the q=1 surface). It can be seen, for example, that the barrier at r≃0.73 is not far from the point where q=3/2, although uncertainties in the *q*-profile do not allow one to make a definite identification.

### 3.4. Propagation Paths

Note that Figure 3 shows two branches of propagation. The “slow branch” is indicated by the white dashed line. However, there appears to be a “fast branch”, visible for 3.55<R<3.74 m at lag times τ<0.01 s. In this section, we investigate this issue further.

The transfer entropy $T(r_{\mathrm{ref}},r,\tau )$ specifies the improvement of the prediction of the next sample of the signal x(r,t), based on the knowledge of $x(r_{\mathrm{ref}},t-\tau )$. Hence, it seems reasonable to assume that some kind of “particles” carry this information from $r_{\mathrm{ref}}$ to *r*, taking a time τ to take this step. In the present context, the “particles” would represent heat, rather than actual particles, of course. The latter description is reminiscent of the continuous time random walk [22].

If one interprets the transfer entropy in this framework, the transfer entropy can be associated with the probability distribution for taking a step $\Delta r=r-r_{\mathrm{ref}}$ in time τ, simply by normalizing $T_{r_{\mathrm{ref}}}\left(\Delta r,\tau \right)=T\left(r_{\mathrm{ref}},r,\tau \right)$ by a factor *N*, so that the resulting distribution $p_{r_{\mathrm{ref}}}\left(\Delta r,\tau \right)=T_{r_{\mathrm{ref}}}\left(\Delta r,\tau \right)/N$ is a probability distribution such that its integral over all relevant Δr and τ equals one. One can then concatenate successive steps of a given particle, drawing the values (Δr,τ) of each step randomly from this probability distribution and study the corresponding compound paths. To reduce the computational load somewhat, we will only consider paths that move strictly outward.

The procedure described above is an iterative procedure, and it allows studying the compound paths statistically. Alternatively, one can use a recursive procedure, by applying a threshold to the step probability distribution. The resulting binary distribution then only states which steps (Δr,τ) are allowed and which are not. Subsequently, all allowed compound outward paths can be followed, using a recursive algorithm, and these can again be subjected to a statistical analysis.

**Figure 7:**
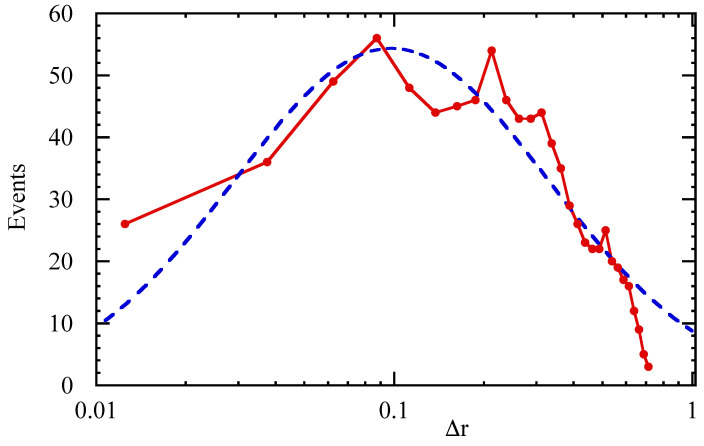
Probability distribution of the radial steps taken by particles, according to the TE analysis. The blue dashed curve is a fitted lognormal distribution.

Figure 7 shows the distribution of radial steps. Previous studies involving the analysis of tracer trajectories in simulations of the topological structures in plasma turbulence suggest that the lognormal distribution may play a significant role [23,24], and indeed, the present result seems to be compatible with this idea, as shown by the fitted line.

**Figure 8:**
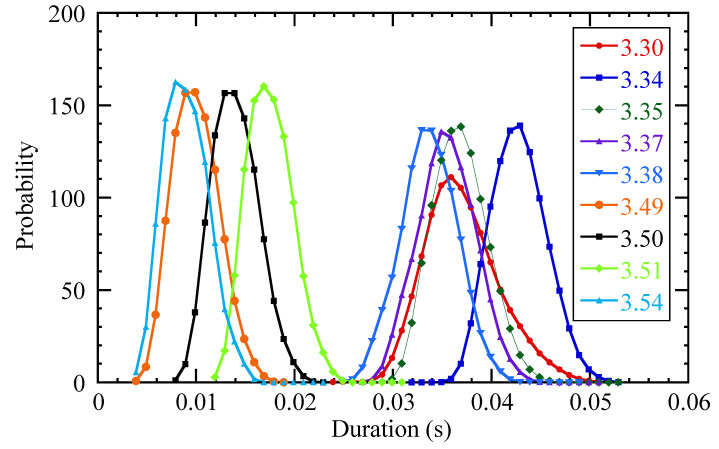
Probability distribution of the times needed to reach the outer edge of the system from an initial position in the core. The legend indicates the position of the first step of the compound path ($R_{2}$).

Figure 8 shows the statistical distribution of the times needed to reach the outer edge of the system from an initial position in the core, calculated from a transfer entropy dataset obtained from ECE data (one element of the set, at a single reference radius, being Figure 3), using the recursive method described above. Remarkably, the distributions seem to separate into two distinct classes, namely fast and slow paths, according to the first step taken ($R_{2}$).

**Figure 9:**
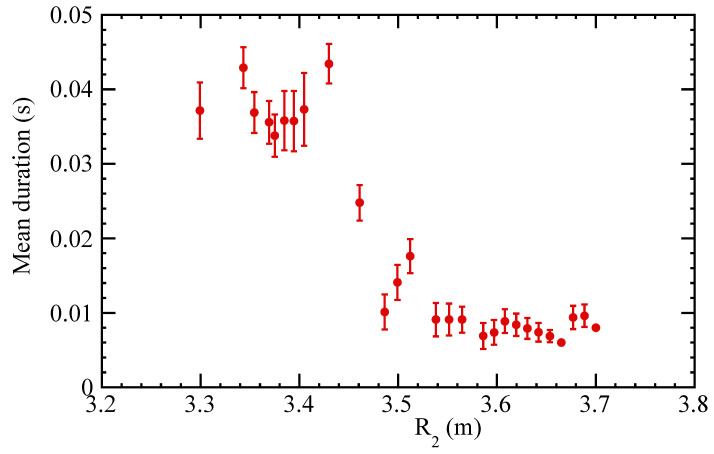
Mean and standard deviation of the durations of the compound paths to reach the edge of the system as a function of the first step taken ($R_{2}$).

The figure shows that each individual distribution is roughly Gaussian, as one might expect. Therefore, these distributions are well characterized by their mean and standard deviation. Figure 9 shows the mean and standard deviation of the durations of the compound paths to reach the edge of the system as a function of the first step taken. The graph separates into two clear classes ($R_{2}<3.45$ and $R_{2}>3.53$), while there is a narrow transition region in-between.

**Figure 10:**
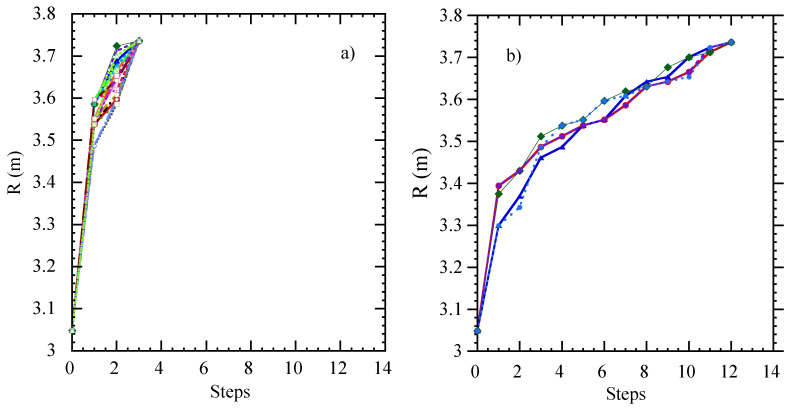
Mean compound paths to reach the edge of the system, for (**a**) paths in the “fast” group and (**b**) paths in the “slow” group of Figure 9.

Figure 10 shows some examples of the fast and slow paths. The slow paths are reminiscent of a directed random walk, while the fast paths include some very long jumps, which suggests they could be Lévy flights [25]. Future work may be able to clarify this point. In any case, the result of this analysis is that radial heat transport in these plasmas appears to be characterized by different transport channels, with different propagation velocities. Presumably, the plasma is able to vary the relative importance of these channels in order to achieve the mentioned self-organization of radial transport.

### 3.5. Modeling

As noted in the Introduction, the plasmas considered here are confined by a magnetic field. Inside the plasma, the magnetic field lines lie on surfaces of constant flux, which have a toroidal topology. The mean field line twist on each surface is such that Δϕ=qΔθ, on average, where Δϕ is the angle in the toroidal direction (long way around the torus) and Δθ is the angle in the poloidal direction (short way around the torus). On each flux surface, *q* is constant. When *q* takes a rational value, the magnetic field lines close on themselves after a finite number of turns. This is where turbulent vortices, which are elongated along the direction of the field line and therefore have a filamentary structure, are preferentially located.

The turbulent flow velocity of the plasma can be expressed as V=b×∇Φ, where Φ is a stream function (proportional to the electrostatic potential) and *b* is a unit vector in the toroidal (field) direction. Theoretically, transport barriers may arise as a consequence of zonal flows generated by turbulence. The mechanics of the interaction between turbulent fluctuations and zonal flows is well understood: fluctuations may generate flows through Reynolds stress [26], and the shear in these flows then suppresses the fluctuations [27]. The complexity of these interactions has been clarified using simplified models [28], and it has been found that sheared flow regions are preferentially formed near rational surfaces.

**Figure 11:**
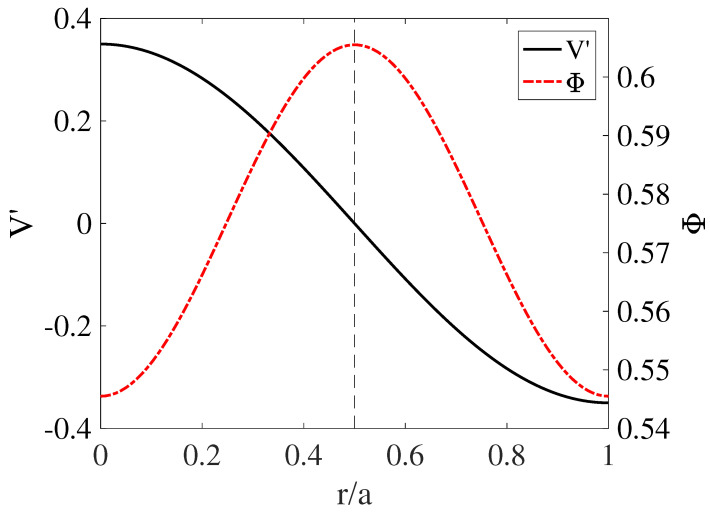
Potential fluctuation (Φ) and shear of the generated flow ($V^{\prime }=dV/dr$) for a simple nonlinear slab model. The vertical dashed line shows the position of the singular surface. Figure reproduced from [15].

Figure 11 shows the radial structure of an electrostatic fluctuation potential near a rational surface, arbitrarily placed at r/a=0.5, and the associated sheared flow in a very simple slab model.

This figure is no more than a cartoon, shown to illustrate the idea of the association between fluctuations, rational surfaces, and sheared flow. If the instability eigenfunction Φ is symmetric with respect to the rational surface, the flow shear $\left|V^{\prime }|=|dV/dr\right|$ will peak off the rational surface, at a distance of the order of the width of the turbulent vortices. Likewise, an antisymmetric eigenfunction will place the flow shear peak at the rational surface. Each type of instability will generate its own structure, possibly modulated by the presence of other structures nearby, and the actual situation can be rather convoluted. Nevertheless, the central idea is that the sheared flow regions are usually located near singular surfaces.

The plasma is pervaded by many types of instability. However, the fact that we detect minor transport barriers associated with rational surfaces provides a hint with regard to the underlying mechanism. Therefore, we have turned to a resistive MHD turbulence model to interpret experimental results [29]. Thus, we have been able to show that the spontaneously arising turbulence in this model generates sheared flow regions that act as minor transport barriers [30]. Injecting tracers to better understand the effect of the turbulence and the sheared flow regions on transport, we have observed that some of the tracers are trapped in the turbulent vortices, while others, with higher kinetic energies, perform rapid radial excursions, “jumping over” the barriers. As the system is driven more strongly (by increasing heating power levels), on average, tracers are endowed with higher energies, so that more tracers will be able to “jump” the minor barriers. In fact, this is the mechanism we proposed to explain the degradation of confinement in the TJ-II stellarator [13]. Likewise, in the framework of the present study, we observe the existence of minor transport barriers and two classes of “particles”: slow and fast, or “diffusive” and “jumping”, which seems to fit nicely with these ideas.

**Figure 12:**
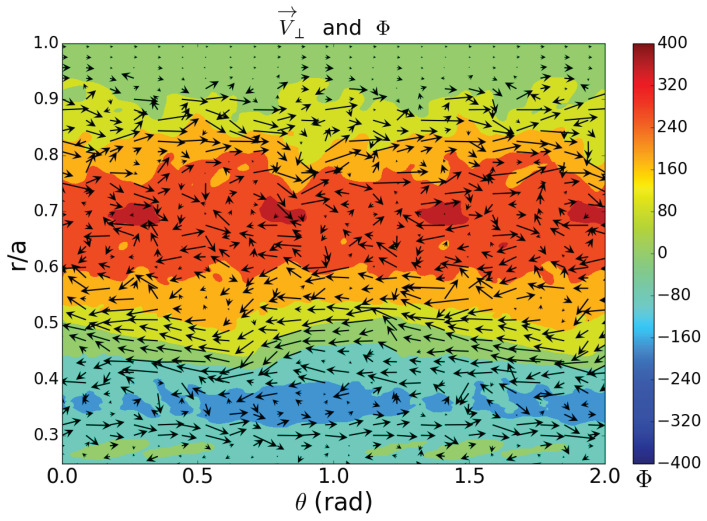
Modeling results, showing zonal flow regions and vortices.

Figure 12 shows a snapshot of a typical modeling result obtained with the mentioned resistive MHD model in stellarator-like (low shear) cylindrical geometry. The area of the graph corresponds to a region of the poloidal-radial (θ,r) plane at constant toroidal angle (ϕ= constant). The graph shows vortices (trapping regions), such as the poloidally periodic structures seen near r/a=0.7, related to a corresponding rational surface. Also visible are zonal flow regions (horizontally elongated structures with predominantly horizontal flow velocities in both directions), on both sides of the vortex sequence.

In previous work, we have successfully applied the transfer entropy to turbulence simulations of this type. This effort yielded a qualitatively similar picture as the reported experimental results, with “trapping zones” and radial “jumps” [10,11]. We also verified the calculation of the effective diffusivity from the TE and compared it to traditional estimates for such simulations [31].

## 4. Discussion

It has long been known that magnetically confined plasmas occasionally develop spontaneous transport barriers. Early work carried out at the RTP tokamak clearly demonstrated the existence of a multiplicity of such transport barriers throughout the plasma, whose location was found to be close to low order rational surfaces [32]. Subsequently, a simplified so-called “*q*-comb” transport model was developed to interpret the observations, based on radially localized reductions of the heat diffusion coefficient, coinciding with low order rational surfaces [33]. However, this and similar work has not led to a general incorporation of mechanisms associated with rational surfaces in heat transport models for fusion plasmas, probably due to the fact that further experimental evidence for these minor transport barriers, associated with rational surfaces, has been difficult to obtain.

Under specific conditions, plasmas can also develop so-called Internal Transport Barriers (ITBs) [34], which arise only transiently, but are much stronger than the “minor transport barriers” that are the focus of this paper. In tokamaks, strong ITBs can be established by creating a core reversed magnetic shear region, while the location of the ITB appears correlated with integral values of the safety factor, *q* [35]. The impact of ITBs on heat transport has been studied in some detail at, e.g., Alcator C-Mod [36] and JET [19,37], showing that the heat diffusivity drops strongly in the ITB region. ITBs have also been obtained and studied in stellarators [38], and here, too, a relationship with the magnetic configuration is suggested. The existence of ITBs is widely acknowledged and supported by experimental evidence on many machines.

A localized transport barrier (i.e., a local reduction of heat flux) implies a local change of slope of the temperature profile. Given the general turbulent state of the plasma and the prevailing measurement resolution and errors, such rather localized changes of slope are usually not easy to detect. Even with strong ITBs, it is often difficult to delimit the precise location of the ITB, based on the temperature profile alone. Hence, it is not very surprising that minor transport barriers usually go undetected. As a result, many transport models completely ignore their possible existence and do not contemplate any effects that explicitly depend on the rational values of the rotational transform.

In our recent series of papers, using a novel method to detect minor transport barriers based on the transfer entropy, we have tried to show that such barriers occur quite frequently, even in plasmas with no easily discernible “steps” in the temperature profile, and they tend to be associated with low order rational surfaces [10,11,13]. By studying the barriers at different heating power levels, we have been able to observe a change in the characteristics of transport (an increased importance of heat “jumping” over the minor barriers) that suggests that these minor barriers could in fact play a prime role in the understanding of the important and ubiquitous phenomenon of power degradation.

To recall, power degradation is the phenomenon that the energy confined in the plasma (*W*) increases less than linearly with the heating power. In all magnetic confinement devices where the scaling of the energy confinement time ($\tau_{E}=W/P$, subject to some caveats and corrections) with heating power (*P*) has been studied, it is found that it scales like $\tau_{E}\propto P^{\alpha_{P}}$, where $\alpha_{P}=-0.6\pm 0.1$ [39,40,41,42,43]. The fact that this scaling holds across the board for the main types of magnetic fusion devices (tokamaks and stellarators) indicates that it must be due to a very basic mechanism, common to these devices.

Our analysis suggests that transport does not involve a single mechanism, but various competing mechanisms, whose relative importance depends on the drive. Hence, describing transport via a single diffusion coefficient (or a similar simplified description) may not be adequate to capture the physics underlying power degradation.

In previous work, we have made use of a resistive MHD model [29] to understand both the detected minor transport barriers and the “jumping” behavior [10,11,13]. While this model does not capture all details of turbulence in fusion-grade plasmas, it does allow a precise analysis of the effect of MHD-type turbulence, which typically is associated with low order rational surfaces. In view of the fact that our analyses seem to indicate that low order rational surfaces play an important role, it makes sense to use this type of model to gain further insight. The modeling results seem to indicate that sheared flow layers tend to form near low order rational surfaces as a consequence of plasma self-organization. These sheared flow layers tend to suppress turbulence locally, leading to minor transport barriers [2]. Near these barriers, turbulent vortices form where radially propagating “particles” can get trapped. The observed “jumping” behavior is also reproduced by the modeling results and could be associated with the coupling between MHD turbulence associated with different rational surfaces or, more generally, “avalanches”. The observations indicate that the “jumping” behavior increases in intensity when the heating power is increased, suggesting an explanation for the phenomenon of power degradation mentioned in the Introduction.

We note that the suggested association with low order rational surfaces may apply only under specific circumstances (namely, those where the resistive MHD model we used are relevant; typically, stellarators). Recent theoretical [44] and experimental [45] work on tokamaks suggests the existence of a so-called E×B “staircase” in hot plasmas, largely analogous to the ideas we propose here, but only loosely connected to rational surfaces, if at all. We conclude from this work that magnetically confined fusion plasmas have a general tendency to self-organize by forming sheared flow layers and minor transport barriers, with characteristics that may depend somewhat on the underlying turbulence mechanisms.

In previous work, we have studied transport from the particle perspective by injecting tracer particles in the turbulent flow computed using the mentioned resistive MHD model [23,24,30,46]. Depending on the energy of the tracer particles, some are trapped by the turbulent vortices, while others, typically with more energy, escape the vortices and end up in the zonal flow regions near the vortices, which constitute a barrier for radial transport. Only particles with the highest energies are able to jump over the barriers [30]. These tracer particle dynamics are consistent with the dynamical picture offered by the transfer entropy analysis presented here.

## 5. Conclusions

This work highlights the non-linear and complex nature of heat transport in strongly driven fusion plasmas. Using a relatively novel analysis method, the transfer entropy, we have shown that heat transport in magnetic fusion devices exhibits qualitatively similar properties in two stellarators and one tokamak. Analysis based on the use of the transfer entropy demonstrates the existence of radially localized zones that can be described as “minor barriers” and associated “trapping regions”. A measure was introduced to quantify the “persistence” of local radial TE minima, associated with the minor barriers. We also devised a simple technique to obtain a crude estimate of the effective local heat diffusivity from the TE. The resulting effective heat diffusivity was found to be compatible with traditional estimates, while showing radial variations that appear to be associated with the previously identified minor barriers.

In previous work on two stellarators, we found that the “minor barriers” appear to be associated with low order rational surfaces. In the tokamak case, the relation with low order rational surfaces was less clear [15]. Heat transport was found to be able to “jump over” these minor barriers to some degree, and as heating power was raised, the “jumping behavior” was shown to increase in intensity [11,13,15], providing a possible explanation for the ubiquitous phenomenon of “power degradation” observed in magnetically confined fusion plasmas.

In the present work, we have extended the analysis by reinterpreting the transfer entropy in terms of a continuous time random walk. This approach revealed the existence of clearly separated “fast” and “slow” transport channels (which also appears to be in accordance with a recent more traditional analysis reported in [47]). We interpret the “slow” channel in terms of the usual diffusive transport, whereas the “fast channel” would be associated with the “jumping” behavior mentioned above. In terms of CTRW terminology, the former would be associated with the standard random walk, whereas the latter would correspond to Lévy walks.

The methodology used here does not allow making quantitative statements about the relative importance of the “fast” and “slow” transport channels. This important issue is left to future work, as is the question of particle transport (as compared to heat transport). Furthermore, so far, we have focused on fusion plasmas with relatively low heating power (L-mode plasmas), the reason being that it is often easier to obtain a steady state in L-mode, while the absence of violent instabilities associated with the H-mode edge transport barrier (so-called edge localized modes) further facilitates the analysis. It is clear, however, that it would be important to extend this work also to H-mode plasmas.

## References

[1] Freidberg J. (2007). Plasma Physics and Fusion Energy.

[2] Diamond P., Itoh S.I., Itoh K., Hahm T. (2005). Zonal flows in plasma—A review. Plasma Phys. Control. Fusion.

[3] Sánchez R., Newman D., Carreras B. (2001). Mixed SOC diffusive dynamics as a paradigm for transport in fusion devices. Nucl. Fusion.

[4] International Fusion Research Council (2005). Status report on fusion research. Nucl. Fusion.

[5] Hutchinson I. (2002). Principles of Plasma Diagnostics.

[6] Hartfuß H.J., Geist T. (2013). Fusion Plasma Diagnostics With Mm-Waves: An Introduction.

[7] Schreiber T. (2000). Measuring information transfer. Phys. Rev. Lett..

[8] van Milligen B., Birkenmeier G., Ramisch M., Estrada T., Hidalgo C., Alonso A. (2014). Causality detection and turbulence in fusion plasmas. Nucl. Fusion.

[9] van Milligen B., Carreras B., García L., Martin de Aguilera A., Hidalgo C., Nicolau J., The TJ-II Team (2016). The causal relation between turbulent particle flux and density gradient. Phys. Plasmas.

[10] van Milligen B., Nicolau J., García L., Carreras B., Hidalgo C., The TJ-II Team (2017). The impact of rational surfaces on radial heat transport in TJ-II. Nucl. Fusion.

[11] van Milligen B., Hoefel U., Nicolau J., Hirsch M., García L., Carreras B., Hidalgo C., The W7-X Team (2018). Study of radial heat transport in W7-X using the Transfer Entropy. Nucl. Fusion.

[12] Alejaldre C., Alonso J., Almoguera L., Ascasíbar E., Baciero A., Balbín R., Blaumoser M., Botija J., Brañas B., de la Cal E. (1999). First plasmas in the TJ-II flexible Heliac. Plasma Phys. Control. Fusion.

[13] van Milligen B., Carreras B., Hidalgo C., Cappa Á., The TJ-II Team (2018). A possible mechanism for confinement power degradation in the TJ-II stellarator. Phys. Plasmas.

[14] Wolf R., Ali A., Alonso A., Baldzuhn J., Beidler C., Beurskens M., Biedermann C., Bosch H.S., Bozhenkov S., Brakel R. (2017). Major results from the first plasma campaign of the Wendelstein 7-X stellarator. Nucl. Fusion.

[15] van Milligen B., Carreras B., de la Luna E., Solano E.R., The JET Team (2018). Radial variation of heat transport in L-mode JET discharges. Nucl. Fusion.

[16] Fredrickson E., Callen J., McGuire K., Bell J., Colchin R., Efthimion P., Hill K., Izzo R., Mikkelsen D., Monticello D. (1986). Heat pulse propagation studies in TFTR. Nucl. Fusion.

[17] Lopes Cardozo N. (1995). Perturbative transport studies in fusion plasmas. Plasma Phys. Control. Fusion.

[18] Mantica P., Ryter F. (2006). Perturbative studies of turbulent transport in fusion plasmas. Comptes Rendus Physique.

[19] Mantica P., Gorini G., Imbeaux F., Kinsey J., Sarazin Y., Budny R., Coffey I., Dux R., Garbet X., Garzotti L. (2002). Perturbative transport experiments in JET low or reverse magnetic shear plasmas. Plasma Phys. Control. Fusion.

[20] Brix M., Hawkes N.C., Boboc A., Drozdov V., Sharapov S.E., JET-EFDA Contributors (2008). Accuracy of EFIT equilibrium reconstruction with internal diagnostic information at JET. Rev. Sci. Instrum..

[21] Soler M., Callen J. (1979). On measuring the electron heat diffusion coefficient in a tokamak from sawtooth oscillation observations. Nucl. Fusion.

[22] Klafter J., Blumen A., Shlesinger M. (1987). Stochastic pathway to anomalous diffusion. Phys. Rev. A.

[23] Carreras B., García L., Llerena I. (2010). Tracer particle trapping times in pressure-gradient-driven turbulence in toroidal geometry and their connection to the dynamics of large-scale cycles. Plasma Phys. Control. Fusion.

[24] García L., Llerena Rodríguez I., Carreras B. (2015). Width and rugosity of the topological plasma flow structures and their relation to the radial flights of particle tracers. Nucl. Fusion.

[25] Balescu R. (2005). Aspects of Anomalous Transport in Plasmas.

[26] Carreras B., Lynch V., García L. (1991). Electron diamagnetic effects on the resistive pressure-gradient-driven turbulence and poloidal flow generation. Phys. Plasmas.

[27] Biglari H., Diamond P., Terry P. (1990). Influence of sheared poloidal rotation on edge turbulence. Phys. Plasmas.

[28] Diamond P., Liang Y.M., Carreras B., Terry P. (1994). Self-regulating shear flow turbulence: a paradigm for the L to H transition. Phys. Rev. Lett..

[29] García L., Carreras B., Lynch V., Pedrosa M., Hidalgo C. (2001). Sheared flow amplification by vacuum magnetic islands in stellarator plasmas. Phys. Plasmas.

[30] García L., Carreras B., Llerena L. (2017). Relation of plasma flow structures to passive particle tracer orbits. Nucl. Fusion.

[31] Nicolau J.H., García L., Carreras B.A., van Milligen B. (2018). Applicability of transfer entropy for the calculation of effective diffusivity in heat transport. Phys. Plasmas.

[32] Lopes Cardozo N., Hogeweij G., de Baar M., Barth C., Beurskens M., De Luca F., Donné A., Galli P., van Gelder J., Gorini G. (1997). Electron thermal transport in RTP: filaments, barriers and bifurcations. Plasma Phys. Control. Fusion.

[33] Schilham A., Hogeweij G., Lopes Cardozo N. (2001). Electron thermal transport barriers in RTP: experiment and modelling. Plasma Phys. Control. Fusion.

[34] Wolf R. (2003). Internal transport barriers in tokamak plasmas. Plasma Phys. Control. Fusion.

[35] Joffrin E., Challis C., Conway G., Garbet X., Gude A., Günter S., Hawkes N., Hender T., Howell D., Huysmans G. (2003). Internal transport barrier triggering by rational magnetic flux surfaces in tokamaks. Nucl. Fusion.

[36] Wukitch S., Boivin R., Bonoli P., Fiore C., Granetz R., Greenwald M., Hubbard A., Hutchinson I., In Y., Irby J. (2002). Double transport barrier experiments on Alcator C-Mod. Phys. Plasmas.

[37] Marinoni A., Mantica P., Eester D.V., Imbeaux F., Mantsinen M., Hawkes N., Joffrin E., Kiptily V., Pinches S.D., Salmi A. (2006). Analysis and modelling of power modulation experiments in JET plasmas with internal transport barriers. Plasma Phys. Control. Fusion.

[38] Fujisawa A. (2002). Transport barriers and bifurcation characteristics in stellarators. Plasma Phys. Control. Fusion.

[39] Stroth U., Murakami M., Dory R., Yamada H., Okamura S., Sano F., Obiki T. (1996). Energy confinement scaling from the international stellarator database. Nucl. Fusion.

[40] Carreras B. (1997). Progress in anomalous transport research in toroidal magnetic confinement systems. IEEE Trans. Plasma Sci..

[41] Doyle E., Houlberg W., Kamada Y., Mukhovatov V., Osborne T., Polevoi A., Bateman G., Connor J., Cordey J., Fujita T. (2007). Chapter 2: Plasma confinement and transport. Nucl. Fusion.

[42] Dinklage A., Maaßberg H., Preuss R., Turkin Y.A., Yamada H., Ascasibar E., Beidler C., Funaba H., Harris J.H., Kus A. (2007). Physical model assessment of the energy confinement time scaling in stellarators. Nucl. Fusion.

[43] Hirsch M., Baldzuhn J., Beidler C., Brakel R., Burhenn R., Dinklage A., Ehmler H., Endler M., Erckmann V., Feng Y. (2008). Major results from the stellarator Wendelstein 7-AS. Plasma Phys. Control. Fusion.

[44] Dif-Pradalier G., Hornung G., Garbet X., Gendrih P., Grandgirard V., Latu G., Sarazin Y. (2017). The *E* × *B* staircase of magnetised plasmas. Nucl. Fusion.

[45] Hornung G., Dif-Pradalier G., Clairet F., Sarazin Y., Sabot R., Hennequin P., Verdoolaege G. (2017). *E* × *B* staircases and barrier permeability in magnetised plasmas. Nucl. Fusion.

[46] Carreras B., Lynch V., Zaslavsky G. (2001). Anomalous diffusion and exit time distribution of particle tracers in plasma turbulence model. Phys. Plasmas.

[47] van Berkel M., Vandersteen G., Zwart H., Hogeweij G., Citrin J., Westerhof E., Peumans D., de Baar M. (2018). Separation of transport in slow and fast time-scales using modulated heat pulse experiments (hysteresis in flux explained). Nucl. Fusion.

